# Prognostic value of oxidative stress-related genes in colorectal cancer and its correlation with tumor immunity

**DOI:** 10.1186/s12864-023-09879-0

**Published:** 2024-01-02

**Authors:** Leilei Yang, Chengfeng Fang, Ruili Zhang, Shenkang Zhou

**Affiliations:** Department of Gastrointestinal Surgery, Key Laboratory of Minimally Invasive Techniques & Rapid Rehabilitation of Digestive System Tumor of Zhejiang Province (Taizhou Hospital of Zhejiang Province affiliated to Wenzhou Medical University), No. 150, Ximen Street, Linhai, Taizhou, 317000 Zhejiang China

**Keywords:** Colorectal cancer, Oxidative stress, Risk model, Prognosis, Biomarker

## Abstract

Oxidative stress (OS) plays an essential role in chronic diseases such as colorectal cancer (CRC). In this study, we aimed to explore the relation between oxidative stress-related genes and CRC prognosis and their involvement in the immune microenvironment. Totally 101 OS-related genes were selected from the MsigDB database. Then, univariate Cox regression was used to explore the prognostic value of the selected genes correlated with the CRC patient survival in the TCGA database. A total of 9 prognostic OS-related genes in CRC were identified. Based on consensus clustering, CRC patients were then categorized into two molecular subtypes. A prognostic risk model containing 8 genes was established using Lasso regression, and CRC patients were divided into high or low-risk groups based on the median risk scores. The predictive value of the 8 genes in CRC prognosis was validated using ROC curves, which indicate that CTNNB1, STK25, RNF112, SFPQ, MMP3, and NOL3 were promising prognostic biomarkers in CRC. Furthermore, the immune cell infiltration levels in different risk groups or CRC subtypes were analyzed. We found that the high-risk or C1 subtype had immunosuppressive microenvironment, which might explain the unfavorable prognosis in the two groups of CRC patients. Additionally, functional experiments were conducted to investigate the effects of OS-related genes on CRC cell proliferation, stemness, and apoptosis. We found that CTNNB1, HSPB1, MMP3, and NOL3 were upregulated in CRC tissues and cells. Knockdown of CTNNB1, HSPB1, MMP3, and NOL3 significantly suppressed CRC cell proliferation, stemness and facilitated CRC cell apoptosis. In conclusion, we established prognostic CRC subtypes and an eight-gene risk model, which may provide novel prognostic indicators and benefit the design of individualized therapeutic strategies for CRC patients.

**Supplementary Information:**

The online version contains supplementary material available at 10.1186/s12864-023-09879-0.

## Introduction

Colorectal cancer (CRC) is the third most frequently diagnosed malignancy, with over 1.9 million new cases reported worldwide in 2020, accounting for 10% of global cancer incidence [1]. Risk factors such as family history, cigarette smoking, excessive drinking, and colonic microbiota infection are associated with the CRC development [2]. Despite the diagnostic and therapeutic advancements, the prognosis in patients diagnosed at advanced stages remains unsatisfactory [3], with the 5-year survival of 65%, and the 10-year survival of 58% in CRC patients [4]. Therefore, it is clinically imperative to identify potential biomarkers for early diagnosis and prognosis evaluation in CRC patients.

Oxidative stress (OS) is regarded as an imbalance in the reactive oxygen species (ROS) production and elimination. Studies have demonstrated that OS is implicated in the pathological processes of different malignancies, including CRC [5, 6]. ROS is produced as a byproduct in mitochondria biogenesis and normal metabolism of oxygen, and participates in the cellular signaling transduction or induction of intracellular defense [7, 8]. The counteractive effects of ROS, such as ROS favoring the proliferation of cancer cells or causing cancer cell death due to excessive ROS production, have been noted, suggesting the tumor-suppressing or tumor-promoting role of ROS in cancer development [8]. Indexes related to OS are clinically useful in the prognosis evaluation of CRC patients [9, 10]. Moreover, multiple studies have demonstrated the many OS-related genes are potential prognostic biomarkers in cancer treatment [11–13]. For example, Xu Wang et al. have identified 34 OS- and ferroptosis-associated genes of predictive value in CRC patient prognosis with good efficacy [14]. Zilu Chen et al. have established a prognostic model for CRC with 14 OS-related genes with high predictive value [15].

ROS is implicated as an important signaling molecule in the tumor microenvironment (TME), which consists of macrophages, immune cells, endothelial cells, fibroblasts, tumor cells and an extracellular matrix (ECM) [16, 17]. ROS has been shown to regulate tumor immunity by mediating the functions of tumor-infiltrating immune cells, including tumor-associated macrophages and regulatory T cells [17, 18]. Thus, it is reasonable to explore the correlation between OS-related transcripts and the CRC immunity, which may provide promising therapeutic targets for the improvement of immunotherapeutic effects in CRC patients. In this study, we intended to identify OS-related genes with prognostic value in CRC patients, sub-classify CRC patients and constructed a prognostic risk model based on the prognostic OS-related genes. The immune cell infiltration levels in different CRC subtypes or risk groups were analyzed using bioinformatics tools. Furthermore, functional assays were conducted to explore the expression and biological functions of selected OS-related genes in CRC. The findings could fill in the potential gap in the knowledge of ROS biology in CRC and might help to develop novel prognostic biomarkers for CRC treatment.

## Material and methods

### Data collection and processing

**Fig. 1:**
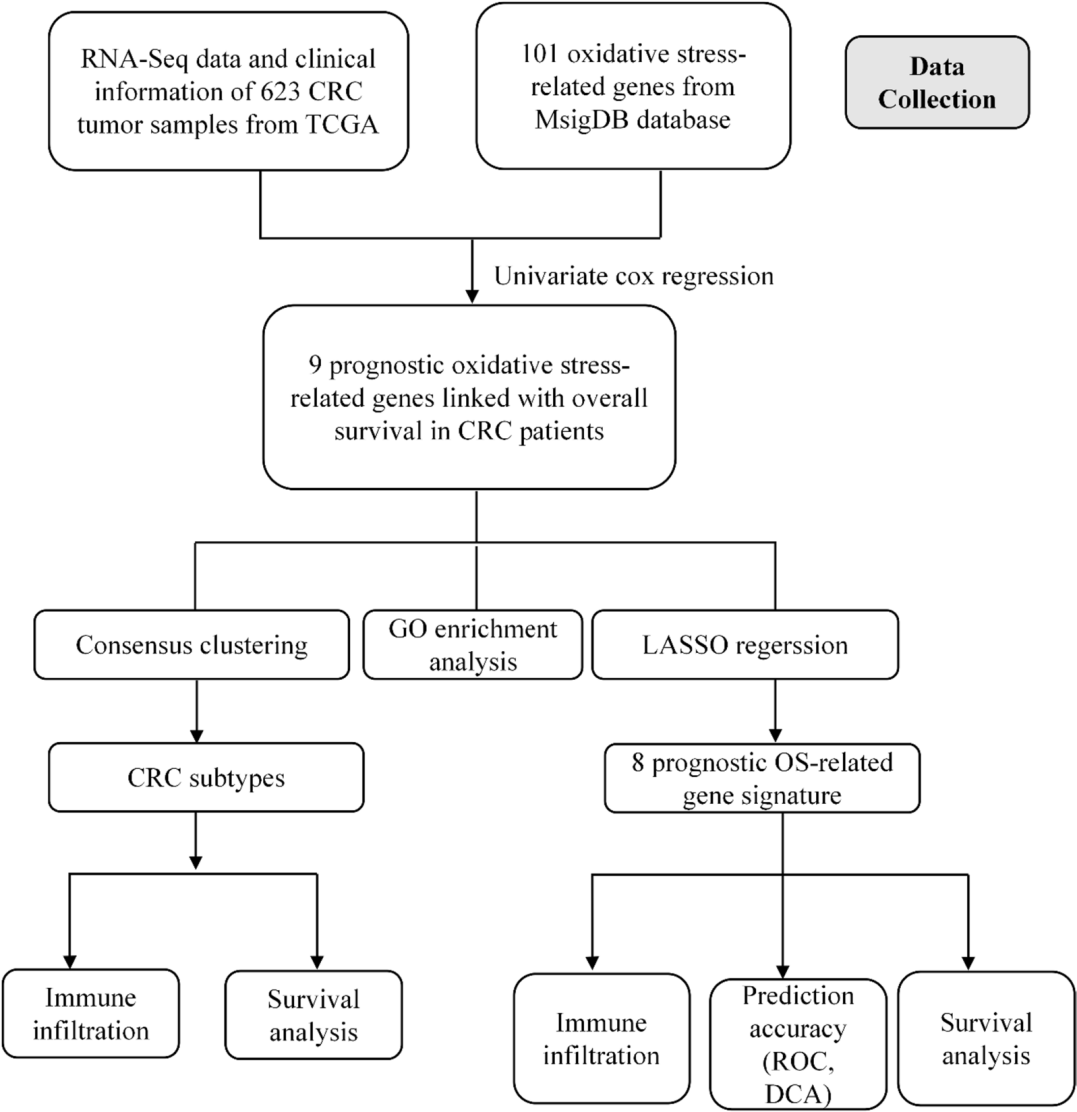
Flow diagram of the study

The RNA-Seq data and clinical information of CRC patients were collected from TCGA-COAD and TCGA-READ projects in ‘The Cancer Genome Atlas’ (TCGA) database (https://portal.gdc.cancer.gov/). RNA-seq information in transcripts per million (TPM) format was retrieved and normalized by log2(value + 1) transformation using the R 4.2.1 software. A total of 623 CRC samples were included in the analysis. The clinical information of patients was provided in the Supplementary material. The flow chart of this study is shown in Fig. 1.

### Identification and functional enrichment analyses of prognostic OS-related genes

The oxidative stress-related genes (*n* = 101) were screened from the MsigDB database (http://www.gsea-msigdb.org/gsea/index.jsp). Univariate Cox regression was used to select prognostic OS-related genes correlated with the survival of CRC patients in the TCGA database with *p* < 0.05 as the threshold value, and 9 genes were obtained. To evaluate the biological functions of OS-related genes, bioconductor annotation package org.Hs.eg.db was used for conversions of gene identifiers, and clusterProfiler package (4.4.4) was used for Gene ontology (GO) enrichment analyses in “Homo sapiens” [19]. GO terms include cell component (CC), biological process (BP) and molecular function (MF). Adjusted *p* < 0.05 was set as the threshold value. The relation between the biological and genetic traits was assessed using the MSigDB.

### CRC Subtype classification

Consensus clustering was conducted to classify CRC cases into different subtypes using the CancerSubtypes R package, which integrates the common computational biology methods for the identification of cancer subtypes [20]. The clustering variable k varied from k = 2 to k = 10. The cumulative distribution function (CDF) plot and CDF delta area curves were applied to determine the optimal cluster number and stability. The prognostic OS-related genes were analyzed by consensus clustering algorithm using agglomerative pam clustering upon 1-pearson correlation distances and resampling 80% of the samples for 10 repetitions.

### Construction and assessment of the prognostic risk model

The prognostic OS-related genes were selected with univariate Cox regression and screened for the construction of the prognostic risk model by the LASSO algorithm using the glmnet R package (4.1.7). The risk score of each CRC patient was calculated as previously documented [11]. Risk Score =${\sum }_{i}^{n}{X}_{i}\times {Y}_{i}$ (*X* indicates correlation coefficient between genes and survival, Y indicates expression level of genes). CRC patients were separated into two (high/low) risk groups with the median risk score as the cutoff value. The differences in patient prognosis between the high and low-risk groups were assessed with Kaplan–Meier analysis using log-rank statistical methods. ROC curves and DCA curves were generated to assess the sensitivity and accuracy of the risk model. The time-dependent receiver operating characteristic (ROC) curves (1-, 3-, and 5-year) were generated using the survminer and timeROC package in R software. *P* < 0.05 was used as the threshold value.

### Immune cell infiltration analysis

To explore the levels of immune cell infiltration in CRC, the ssGSEA algorithm in the R GSVA package (1.44.5) was used to analyze the enrichment of 24 immune cells in CRC samples [21]. Then, levels of immune cell infiltration were compared between different CRC subtypes or risk groups based on expression of the marker genes for 24 types of immune cells [22]. ESTIMATE package was used for the calculation of the stromal score, immune score as well as ESTIMATE score of CRC patients in different groups. Pearson’s correlation analysis was conducted to evaluate the relation between Lasso risk score and infiltration levels of immune cells or purity of CRC tumors.

### Predictive value of the selected OS-related genes in CRC prognosis

ROC curves were used to evaluate the value of the 8 OS-related genes to predict CRC prognosis. The pROC and ggplot2 R packages were used to generate ROC curves, with the False Positive Rate (FPR) as x axis and True Positive Rate (TPR) as y axis.

### Clinical specimens

Thirty pairs of CRC tissue samples, as well as adjacent normal tissues were obtained during surgical treatment from patients with CRC at our hospital. The specimens were stored at − 80 °C until further analysis. All participants had received no chemotherapy or radiotherapy prior to the surgery. Written informed consent was signed by all participants, and the study was approved by the Ethics Committee of our hospital.

### Cell culture and cell transfection

Human CRC cell lines (SW480, HT29, HCT116) and embryonic kidney (HEK293T) cells were provided by the American Type Culture Collection (VA, USA). SW480 and HEK293T cells were incubated in DMEM (Thermo Fisher). HT29 and HCT116 cells were cultured in McCoy’s 5A media. Both culture media were supplemented with 10% FBS and 1/100 Penicillin/Streptomycin in a humidified incubator containing 5% CO_2_ at 37 °C. To silence CTNNB1, HSPB1, MMP3 and NOL3, shRNAs targeting the respective genes were obtained from GENESEED Company (Guangzhou, China) and transfected into CRC cells with Lipofectamine™ 3000 in accordance with the manufacturer’s protocol.

### RT-qPCR

Cells were harvested, and total RNA was extracted using TRIzol (Thermo Fisher). Then the collected RNA was reverse transcribed into cDNA using a SuperScript First-Strand Synthesis System. qPCR was subsequently performed with the SYBR Green I dye detection (Takara, Japan) on a real-time detection system (Bio-Rad). Relative RNA expression was quantified with the 2^−ΔΔCt^ method normalized to GAPDH. The sequences of primers are shown in Table 1.

**Table 1:** Sequences of primers used in this study

STK25	Forward: 5’-TGGACTTGCTTAAACCAGG-3’
	Reverse: 5’-GATAATCCAGGCCCTTCAG-3’
CTNNB1	Forward: 5’-CCAAGTCCTGTATGAGTGGG-3’
	Reverse: 5’-GCATACTGTCCATCAATATCAGC-3’
HSPB1	Forward: 5’-CTTCACGCGGAAATACACG-3’
	Reverse: 5’-TGGTGATCTCGTTGGACTG-3’
MMP3	Forward: 5’-GACTCCACTCACATTCTCC-3’
	Reverse: 5’-AAGTCTCCATGTTCTCTAACTG-3’
SFPQ	Forward: 5’-AATGAACATGGGAGATCCCT-3’
	Reverse: 5’-GCTTCATAACCTATGCCACC-3’
RNF112	Forward: 5’-GCCTTGTCAGTCACTTCCT-3’
	Reverse: 5’-GTATGGGACCAACTGTTGC-3’
NOL3	Forward: 5’-TAAAGAGGCTGAACCGGAG-3’
	Reverse: 5’-TTCAGGAATCTTCGGACTCG-3’
PAGE4	Forward: 5’-CCACCAACTGACAATCAGG-3’
	Reverse: 5’-ACCTTCTACTTTACGTTCTTCG-3’
GAPDH	Forward: 5’-CCTCCTGTTCGACAGTCAG-3’
	Reverse: 5’-CATACGACTGCAAAGACCC-3’

### Western blot

Total protein was extracted from the CRC tissue samples using RIPA buffer (Thermo Fisher). The protein samples were separated by the SDS-PAGE gels and then electro-transferred onto polyvinylidene difluoride membranes. Next, the membranes were blocked with 5% nonfat milk for 60 min, probed with anti-CTNNB1, anti-HSPB1, anti-MMP3, and anti-NOL3, and incubated overnight at 4 °C. GAPDH was used as a loading control. The membranes were then incubated with the secondary antibodies for 60 min at room temperature. The blots were visualized using ECL chemiluminescence reagent (Amersham Biosciences) and then quantified using ImageJ software.

### Immunofluorescence

CRC cells were seeded into 24-well plates, immersed in 4% PFM and treated with 0.4%Triton X-100. Next, cells were blocked with 5% bovine serum albumin for 30 min at ambient temperature, and incubated with primary antibody against CTNNB1, HSPB1, MMP3, and NOL3 at 4 °C overnight. Then the cells were cultured with fluorescent secondary antibody in dark for 60 min, followed by staining the cell nuclei with DAPI solution (Sigma-Aldrich, USA). Finally, the images were captured using a fluorescence microscopy (Leica; Wetzlar, Germany).

### Cell proliferation

After plating the transfected CRC cells into six-well plates (5000 cells per well), cells were maintained at 37 °C for two weeks. Then, the colonies of CRC cells were fixated using paraformaldehyde (PFM) and stained using 0.1% crystal violet (Sigma-Aldrich) and the number of colonies was counted manually under a microscope.

### Flow cytometry analysis

Transfected CRC cells were harvested and centrifuged for five minutes at 1500 rpm and washed with 1 × PBS three times. Subsequently, cells were suspended in the binding buffer supplemented with 5 μL of FITC-conjugated Annexin V and cultured for thirty minutes at 4 °C. Then, 5 μL of propidium iodide was added and incubated for five minutes at room temperature. CRC cell apoptosis in each group was evaluated using flow cytometry (Thermo Fisher, Rockford, IL, USA).

### Sphere formation assay

CRC cell stemness was determined using sphere formation assays. Briefly, transfected CRC cells were seeded in ultra-low attachment plates and incubated in 2 ml serum-free DMEM-F12 medium (Thermo Fisher) containing 10 μg/L bFGF (Thermo Fisher), 20 μg/L EGF (Sigma-Aldrich) and B27 (1:50, Thermo Fisher). After two weeks, cells were fixated with PFM and then stained with crystal violet for 15 min. The number of spheres was calculated under a microscope.

### Statistical analysis

R software and GraphPad Prism 8.0 were used for data analysis and visualization. Data values are reported as the mean ± standard deviation. Statistical differences among three or more groups were assessed using one-way Analysis of Variance. The differences were considered statistically significant when the *P* value was less than 0.05.

## Results

### Identification and enrichment analysis of prognostic OS-related genes in CRC

**Fig. 2:**
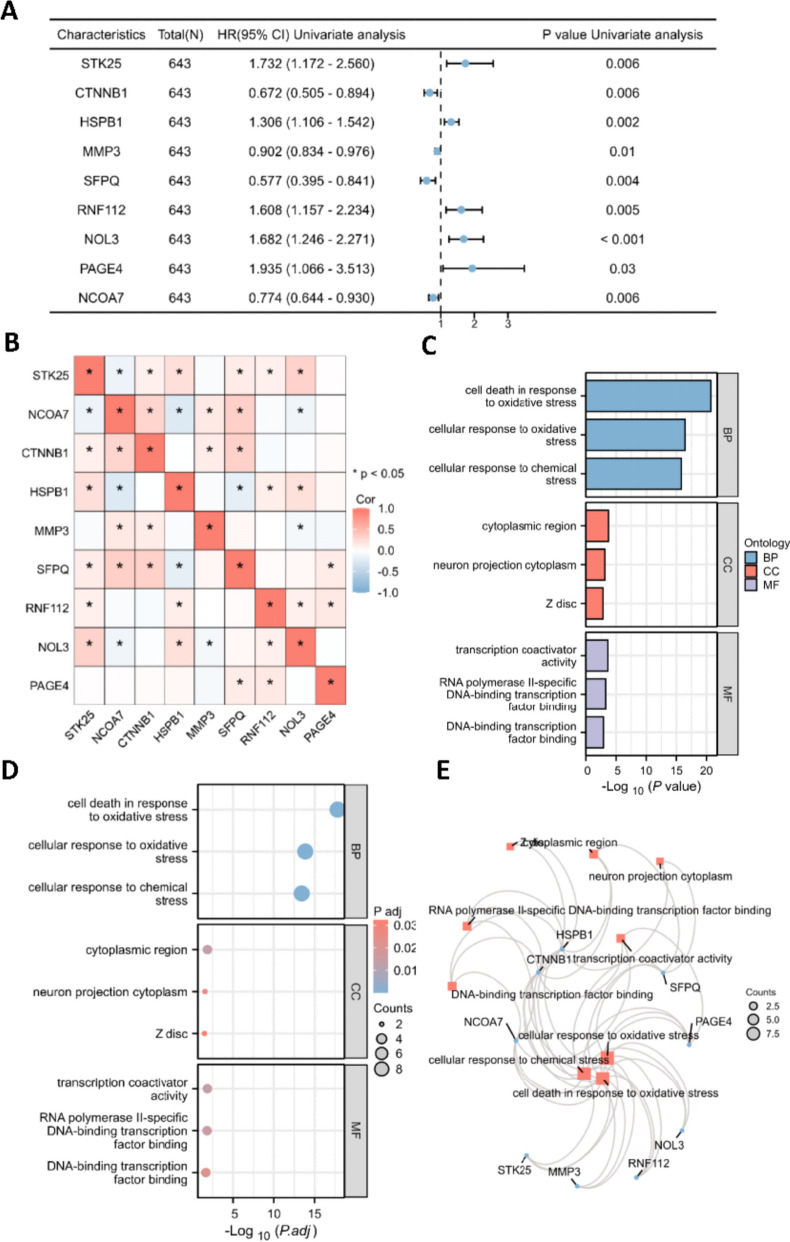
Identification and enrichment analysis of prognostic oxidative stress-related genes in CRC. **A** Univariate Cox regression analysis was conducted to select the prognostic oxidative stress-related genes in CRC. **B** Heatmap of the expression correlation of the 9 selected oxidative stress-related genes. **C** Bar plot and (**D**) Bubble chart of the biological functions of 9 genes based on GO enrichment analysis. **E** Interactions of the GO terms

The prognostic OS-related RNA transcripts in CRC were screened using MsigDB and TCGA databases. Based on MsigDB, we identified 101 oxidative stress-related genes. Then, univariate Cox regression analysis selected 9 prognostic OS-associated genes (STK25, CTNNB1, HSPB1, MMP3, SFPQ, RNF112, NOL3, PAGE4, NCOA7) in CRC based on the relation between genes and overall survival of CRC patients in TCGA database (Fig. 2A). The correlation between the expression of 9 genes in CRC is presented in the heatmap in Fig. 2B, and expression of most genes was significantly correlated in CRC samples. Furthermore, the underlying biological functions of the 9 genes were evaluated by GO analyses. The nine genes were involved in cell death in response to OS, cellular response to OS and chemical stress in terms of biological process (BP); cytoplasmic region, neuron projection cytoplasm and Z disc in terms of cellular component (CC); transcription coactivator activity, RNA polymerase II-specific DNA-binding transcription factor binding and DNA-binding transcription factor binding in terms of molecular function (MF) (Fig. 2C-E).

### Identification of CRC subtypes with OS-related genes

**Fig. 3:**
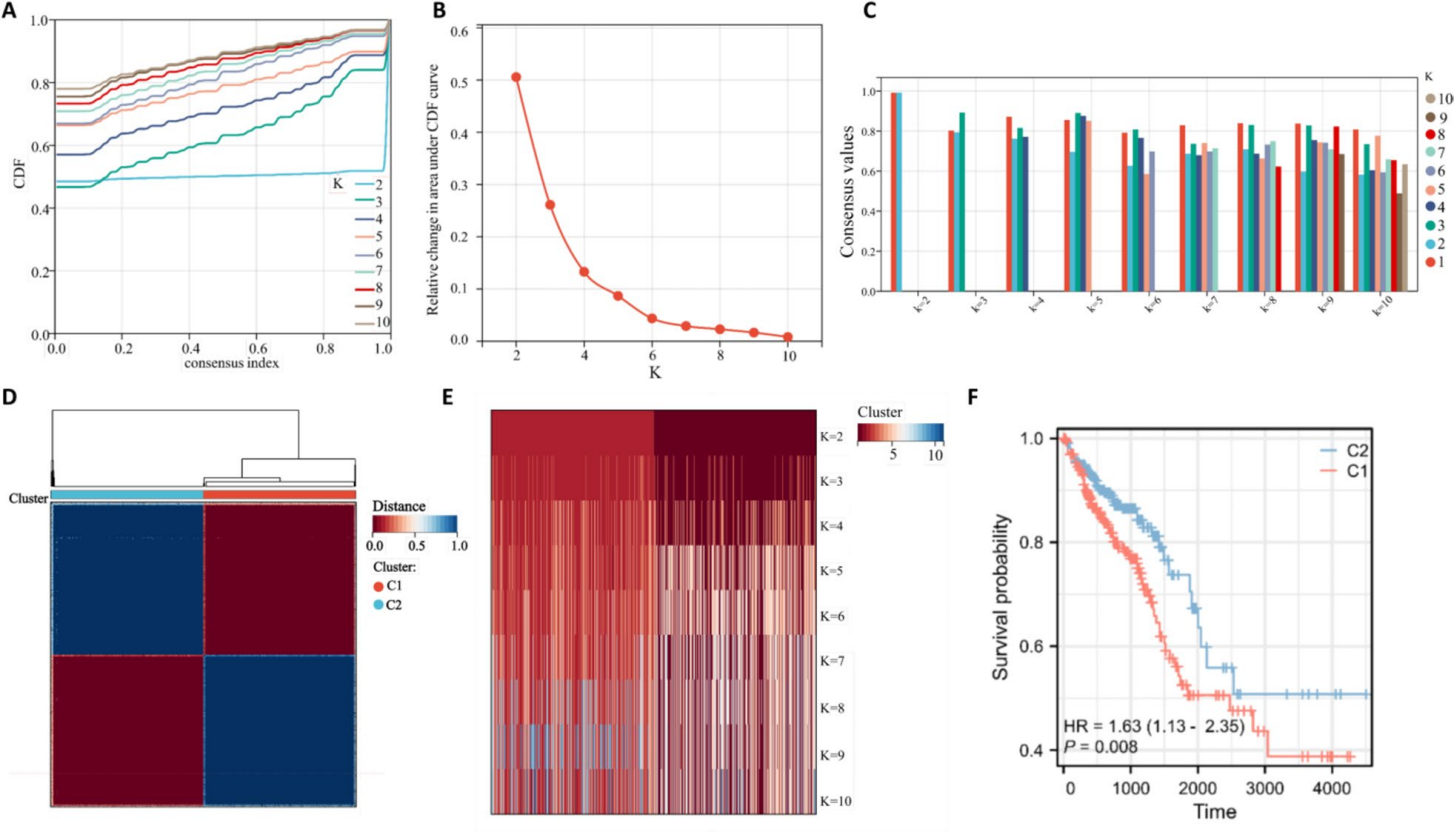
Consensus clustering of CRC subtypes based on oxidative stress-related genes. **A** Consensus clustering model with CDF for k = 2–10. **B** Changes in CDF delta area curve of TCGA for k = 2–10. **C** Bar plot of consensus score in each subgroup with indicated cluster count (2–10). **D** Heat map of sample clustering when k = 2. **E** Tracking plot for k = 2–10 in the TCGA database. **F** Survival outcome in the two subtypes

CRC patients were classified into different molecular subtypes using consensus clustering based on the expression of the 9 prognostic OS-related genes. Cumulative distribution function (CDF) curves and CDF Delta area curves were used to determine the optimal number of clusters. When clustering variable k = 2, comparatively stable clustering results were obtained (Fig. 3A-B), and patients were classified into one of the two OS-related subtypes (C1, C2) (Fig. 3D). Furthermore, we found that only in the 2-subtype classification the cluster consensus score for all subtypes was higher than 0.8, suggesting that the 2-subtype classification was more robust compared with the others (Fig. 3C). Additionally, the heatmap presented the consensus matrix with 2 cluster count and the gene expression profile showed high similarity in each subtype (Fig. 3D). The tracking plot revealed that the samples were distinctly divided into 2 subtypes, which was more robust when k = 2 (Fig. 3E). Then, the prognosis of CRC patients in the two subtypes was evaluated, and the results indicated that patients had a more favorable prognosis in the C2 subtype (Fig. 3F).

### Construction of the prognostic risk model with OS-related genes

**Fig. 4:**
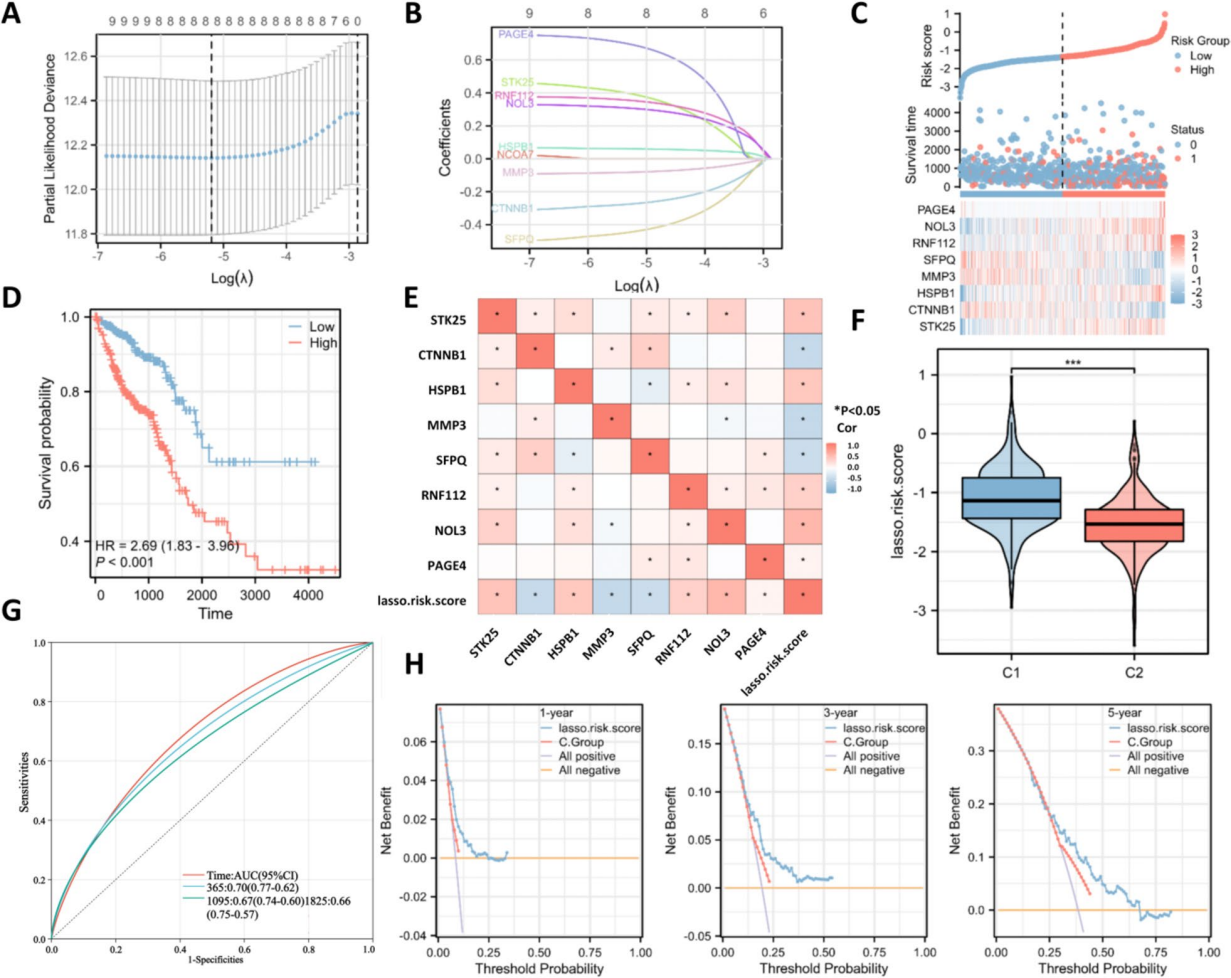
Construction of the prognostic risk model with OS-related genes. **A** The best lambda value was screened by Lasso regression. **B** The coefficient profiles of each oxidative stress-related gene; **C** The risk score, survival outcome, and heatmap of 8 oxidative stress-related genes in CRC patients. **D** Survival curves of CRC patients in indicated groups. **E** Correlation between the levels of 8 oxidative stress-related genes and the Lasso risk score. **F** Profile of the Lasso risk score in the C1 and C2 subtypes. **G** The ROC curves were used to evaluate the accuracy of prognostic risk models. **H** Decision curve analysis (DCA) curves were used to evaluate the net benefits of the models

To construct the OS-related prognostic risk model, Lasso regression was used to analyze the 9 prognostic OS-related genes selected by univariate Cox regression analyses to prevent the model from being overfitted. Eight OS-related genes were selected by Lasso into this prognostic signature, including PAGE4, STK25, RNF112, NOL3, HSPB1, CTNNB1, MMP3 and SFPQ (Fig. 4A-B). We then calculated the risk score of each CRC patient and classified them into high/low-risk groups. The distribution of risk score and expression pattern of the 8 OS-related genes in CRC samples were presented in Fig. 4C. Patients in the high-risk group had a lower survival rate (Fig. 4D). The correlation between the levels of 8 OS-related genes with each other or with the Lasso risk score was presented in Fig. 4E. We also revealed that the C1 CRC subtype had higher Lasso risk scores than the C2 CRC subtype (Fig. 4F). Then ROC curves were generated to evaluate the sensitivity of the prognostic risk model, and the AUCs for the 1-, 3-, and 5-year overall survival were 0.70, 0.67 and 0.66, suggesting the high accuracy of the 8-gene prognostic risk model for predicting prognosis in CRC patients (Fig. 4G). Then, the decision net analysis (DCA) curves were used to evaluate model reliability, and the results showed that the Lasso risk score had better predictive performance and higher value for clinical application (Fig. 4H).

### Prognostic potential of 8 OS-associated genes in CRC

**Fig. 5:**
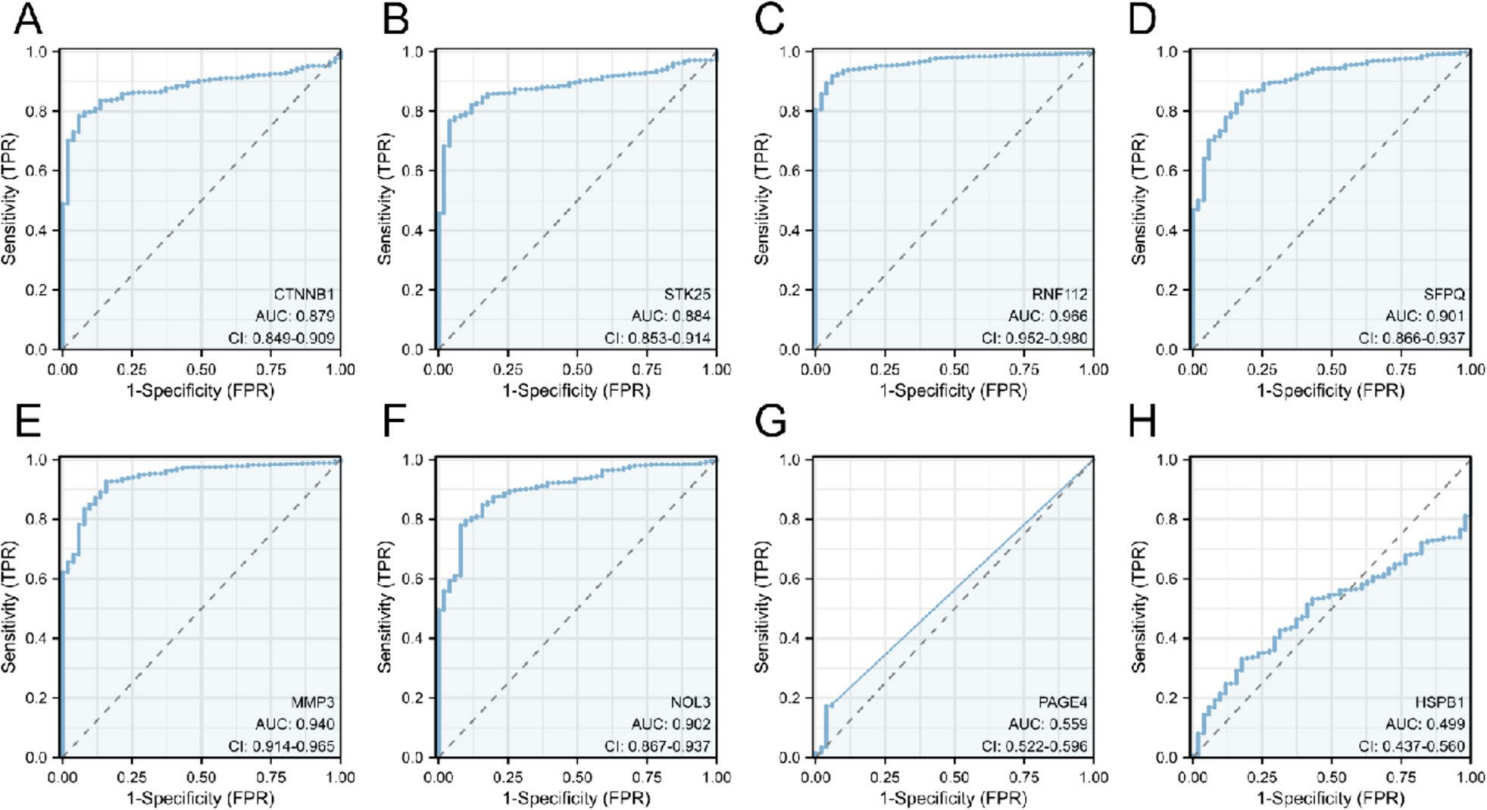
Prognostic potential of 8 OS-associated genes in CRC. ROC curves of the (**A**) CTNNB1, (**B**) STK25, (**C**) RNF112, (**D**) SFPQ, (**E**) MMP3, (**F**) NOL3, (**G**) PAGE4 and (**H**) HSPB1 for the prediction of prognosis in CRC patients

ROC curves were used to evaluate prognostic potential of the 8 OS-associated genes in CRC. The results showed that AUC of CTNNB1 (AUC: 0.879, [0.849–0.909]) (Fig. 5A), STK25 (AUC: 0.884, [0.853–0.914]) (Fig. 5B), RNF112 (AUC: 0.966, [0.952–0.980]) (Fig. 5C), SFPQ (AUC: 0.901, [0.866–0.937]) (Fig. 5D), MMP3 (AUC: 0.940, [0.914–0.965]) (Fig. 5E) and NOL3 (AUC: 0.902, [0.867–0.937]) (Fig. 5F) was over 0.8, showing good performance in the prognosis prediction. The AUC of PAGE4 was 0.559 (0.522–0.596) (Fig. 5G), with a relatively low accuracy, while the AUC of HSPB1 was 0.499 (0.437–0.560) (Fig. 5H), with the lowest prognostic value in CRC among the 8 OS-associated mRNAs.

### Correlation between the infiltration of immune cells with risk score or CRC subtypes

**Fig. 6:**
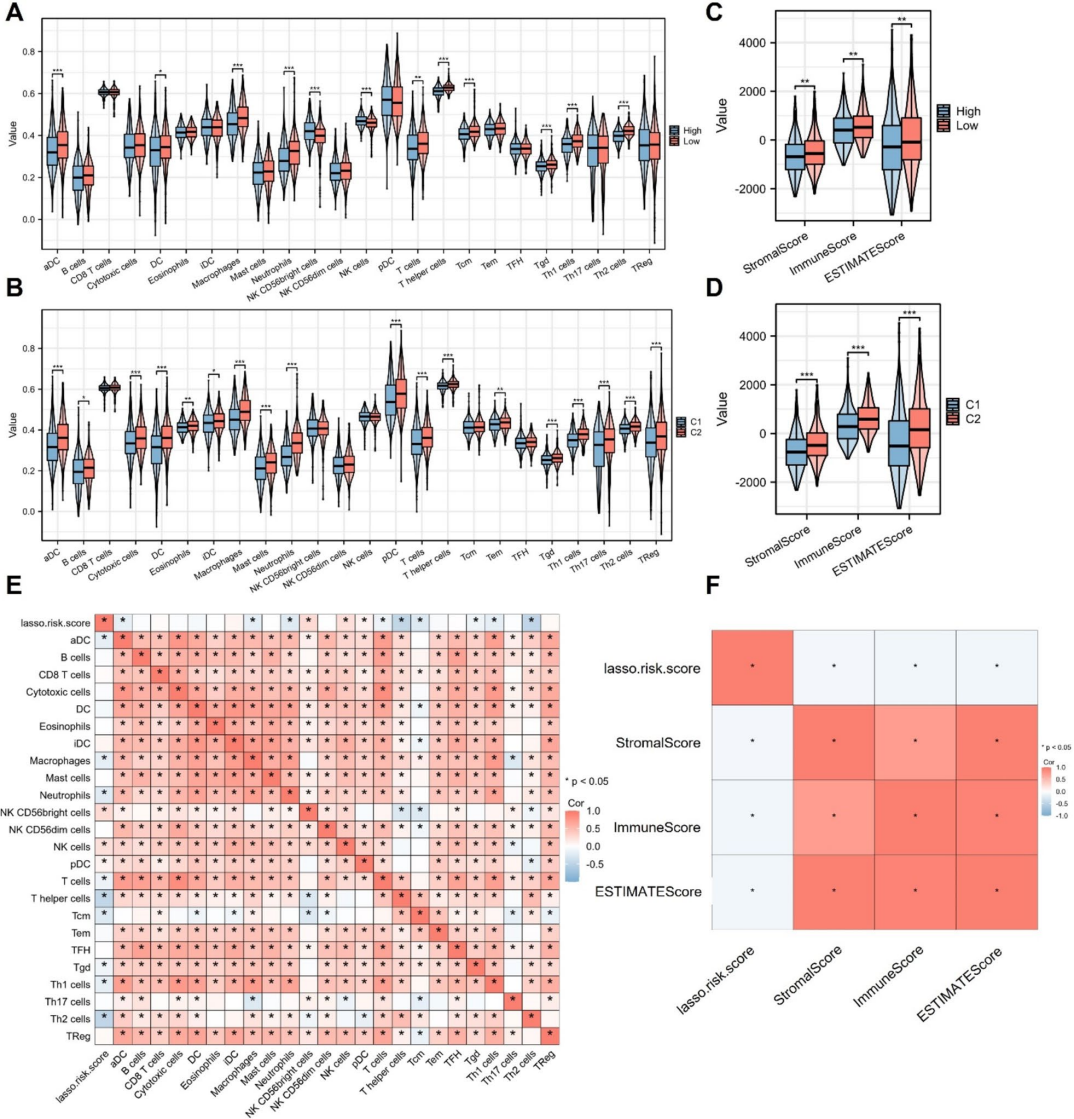
Correlation between risk score or CRC subtype and the tumor immune cell infiltration. **A** ssGSEA was used for the determination of immune cell infiltration in different subtypes or risk groups. **B** ESTIMATE algorithm was applied for the assessment of the tumor purity in indicated groups. **C** Heatmap showed the correlation between the Lasso risk score and the immune cell infiltration levels in CRC patients. **D** Heatmap showed the correlation between the Lasso risk score and the stromal, immune, and ESTIMATE scores

The prognosis of CRC patients is significantly affected by the tumor immune microenvironment [23]. As revealed by ssGSEA, the difference in tumor immunity between two risk groups or between the two CRC subtypes was shown in Fig. 6A-B. The high-risk group showed higher levels of NK CD56bright cells and NK cells, while the CRC patients in the low-risk group showed increased infiltration levels of aDC, DC, macrophages, neutrophils, T cells, T helper cells, Tcm, Tgd, Th1 cells and Th2 cells, which suggested that patients in the low-risk group had stronger tumor immune response relative to the high-risk group, and the infiltration of these immune cells may possibly affect the prognosis of CRC patients. Moreover, those in the C2 CRC subtype showed higher levels of aDC, B cells, cytotoxic T cells, DC, eosinophils, iDC, macrophages, mast cells, neutrophils, pDC, T cells, T helper cells, Tem, Tgd, Th1 cells, Th17 cells, Th2 cells and Tregs, which were also related to the enhanced immune response compared with the C1 CRC subtype. We then assessed the difference in tumor immune microenvironment in different groups. Results demonstrated that CRC patients in the high-risk group or C1 subtype had significantly reduced stromal, immune, and ESTIMATE scores, which indicated that the TME was significantly different between the high/low-risk groups or the C1/C2 subtypes (Fig. 6C-D). As shown in Fig. 6E, the correlation between the Lasso risk score with the immune infiltration levels was explored, and the Lasso risk score was positively related to the immune infiltration levels of NK CD56 bright cells, NK cells and pDCs, and negatively related to the immune infiltration levels of aDCs, macrophages, neutrophils, T cells, T helper cells, Tcm, Tgd, Th1 cells and Th2 cells. Additionally, we also identified the negative correlation between the Lasso risk scores with the immune, ESTIMATE, and stromal scores in CRC, suggesting the association between high risk score and the immunosuppressive tumor microenvironment in CRC patients (Fig. 6F). Overall, these results indicated that the low-risk group or C2 subtype presented a stronger tumor immune response and may benefit from immunotherapy relative to the high-risk group or C1 subtype.

### Association between OS-related genes and CRC stemness and microsatellite instability

**Fig. 7:**
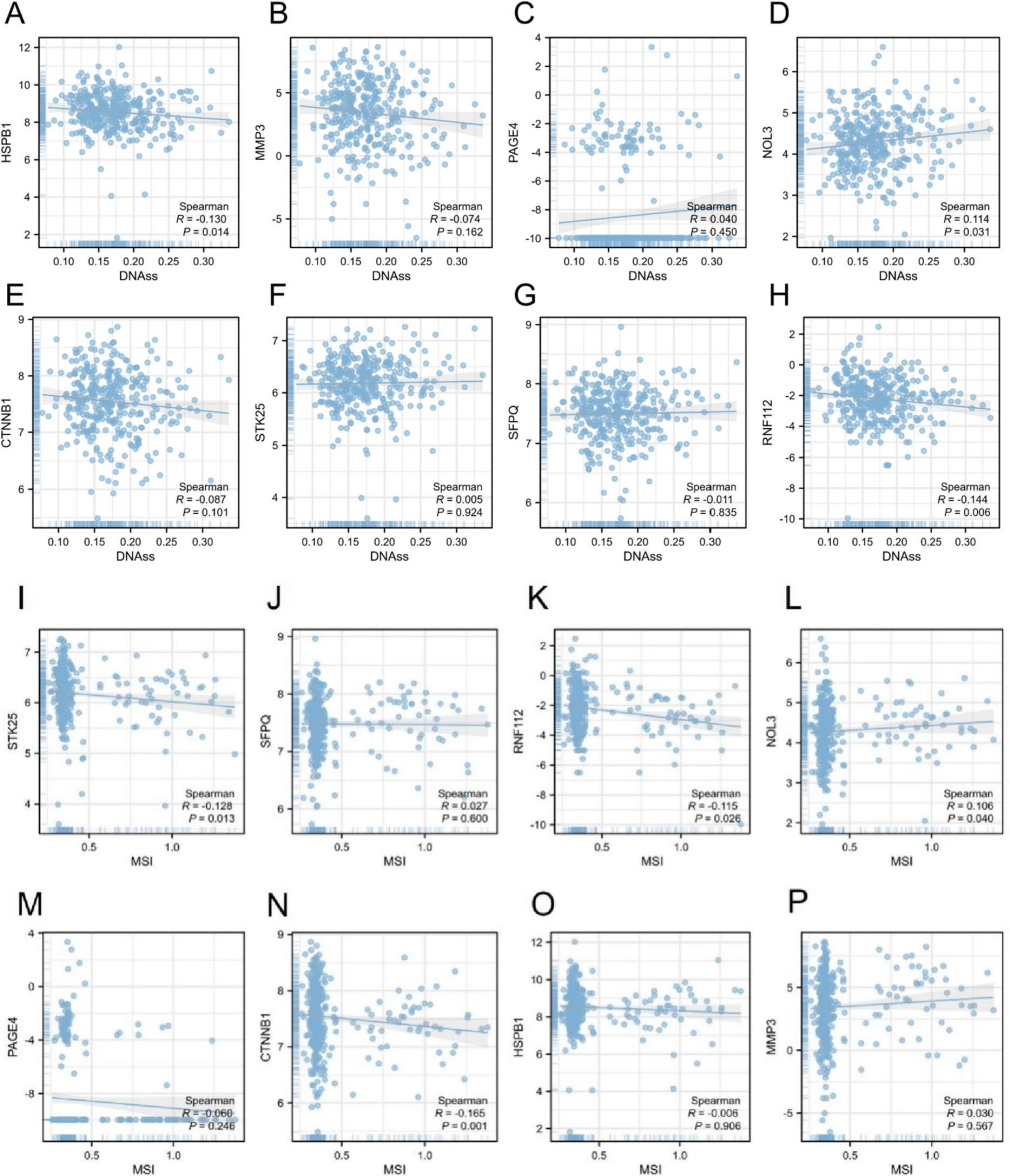
Association between OS-associated genes and CRC stemness and microsatellite instability. The expression correlation of (**A**) HSPB1, (**B**) MMP3, (**C**) PAGE4, (**D**) NOL3, (**E** CTNNB1, (**F**) STK25, (**G**) SFPQ and (**H**) RNF112 with the stemness scores based on DNA methylation was subject to Spearman correlation analysis. The correlation of the expression of (**I**) STK25, (**J**) SFPQ, (**K**) RNF112, (**L**) NOL3, (**M**) PAGE4, (**N**) CTNNB1, (**O**) HSPB1 and (**P**) MMP3 with microsatellite instability (MSI) in CRC patients

ROS is reported to maintain stem cells and is implicated in the modulation of stemness-related properties in cancer progression [24]. Thus, we further analyzed the relation between the eight prognostic OS-related genes and CRC cell stemness. As revealed by Spearman correlation analysis, the levels of HSPB1, MMP3, CTNNB1, SFPQ and RNF112 showed a slight positive association with the stemness scores in CRC, and the levels of PAGE4, NOL3 and STK25 were slightly positively related to the stemness scores in CRC (Fig. 7A-H).

Oxidative stress can cause cellular DNA damage. Microsatellite instability (MSI) is an indicator of chromosome instability and also one of the main oncogenic pathways of CRC. We subsequently analyzed the association between 8 prognostic genes and MSI in CRC. We found that STK25, RNF112, PAGE4, CTNNB1 and HSPB1 expression was negatively correlated with the MSI, while the levels of SFPQ, NOL3 and MMP3 were positively correlated with the MSI, although not significant (Fig. 7I-P).

### Expression pattern of 8 prognostic OS-related genes in CRC

**Fig. 8:**
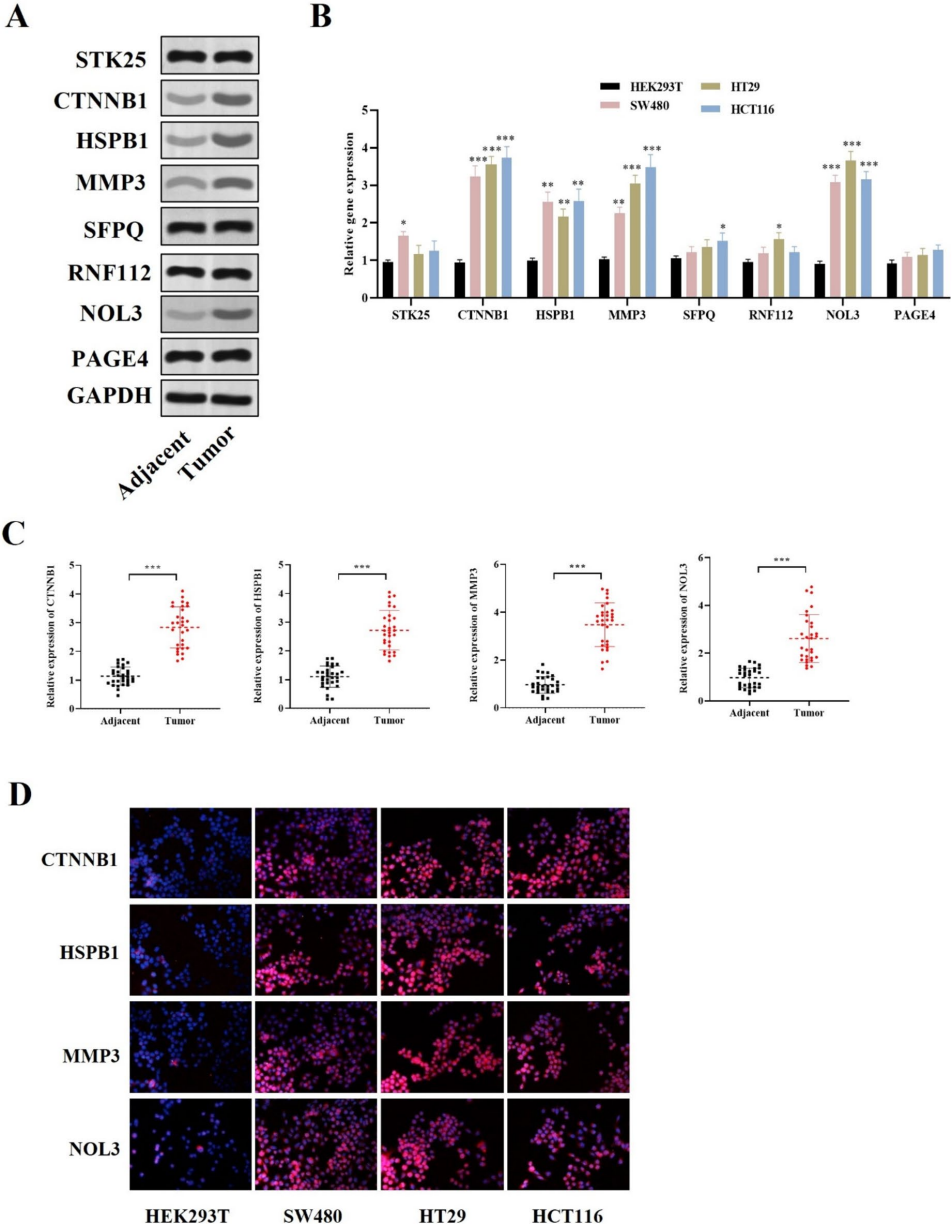
Expression profile of 8 prognostic OS-related genes in CRC. **A** Western blot was conducted to detect the protein expression of the 8 oxidative stress-related genes in CRC tumor specimens and adjacent normal samples. **B** RT-qPCR was used to measure the mRNA levels of the 8 oxidative stress-related genes in CRC cell lines and HEK293T cells. **C** RT-qPCR was used to detect the mRNA expression of the four dysregulated four genes. **D** Immunofluorescence assays showed the upregulation of four dysregulated four genes in CRC cells and HEK293T cells. *P < 0.05, **P < 0.01, ***P < 0.001

We further investigated the mRNA and protein levels of 8 prognostic OS-related genes in CRC patient tissue specimens and cells. The mRNA and protein levels of CTNNB1, HSPB1, MMP3 and NOL3 were significantly upregulated in the tumor samples of CRC patients, and the expression of the other 4 genes showed no significant difference between CRC tumor samples and adjacent normal tissue samples (Fig. 8A-B). Moreover, the RT-qPCR analysis and immunofluorescence assays also indicated the upregulation of CTNNB1, HSPB1, MMP3 and NOL3 mRNAs in all CRC cells (Fig. 8C-D).

### Effects of CTNNB1, HSPB1, MMP3 and NOL3 knockdown on CRC cell proliferation, stemness and apoptosis

**Fig. 9:**
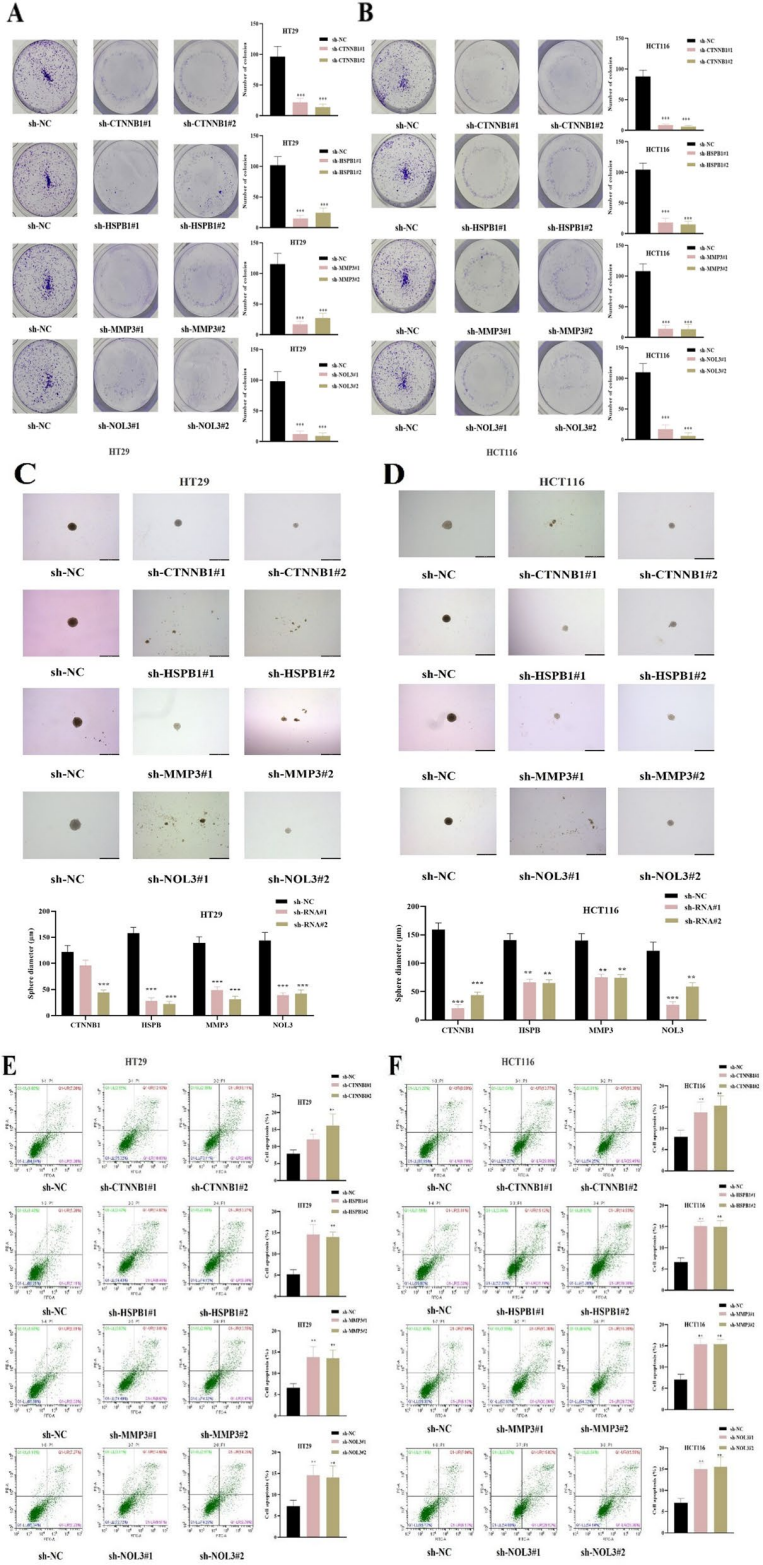
Effects of CTNNB1, HSPB1, MMP3 and NOL3 knockdown on CRC cell proliferation, stemness and apoptosis. **A**-**B** Colony formation assays were performed to assess the proliferation of CRC cells after indicated transfection. **C**-**D** Sphere formation assays for the assessment of CRC cell stemness in each group. **E**–**F** Flow cytometry was used to assess CRC cell apoptosis in each group. *P < 0.05, **P < 0.01, ***P < 0.001

Functional experiments were conducted to evaluate the impact of dysregulated genes (CTNNB1, HSPB1, MMP3, NOL3) on the cellular model of CRC malignancy. We found that silencing of CTNNB1, HSPB1, MMP3 and NOL3 significantly reduced the colony number of CRC cells (Fig. 9A-B). As revealed by the sphere formation assays, the stemness of CRC cells was significantly inhibited after silencing CTNNB1, HSPB1, MMP3 and NOL3 (Fig. 9C-D). On the contrary, the apoptosis rate of CRC cells was elevated after the knockdown of CTNNB1, HSPB1, MMP3 and NOL3 (Fig. 9E-F). Overall, these results indicated that CTNNB1, HSPB1, MMP3 and NOL3 knockdown suppressed the proliferation stemness and promoted the apoptosis of CRC cells.

## Discussion

CRC is the second most fatal malignancy, with around 935,000 deaths cases in 2020 worldwide [1]. Oxidative stress resulting from oxidant/antioxidant imbalance can lead to DNA and protein modification and lipid peroxidation and is closely associated with CRC development [6]. Therefore, the exploration of prognostic oxidative stress-related biomarkers is instrumental to design personalized therapeutic plans and improve the clinical outcome of CRC patients. In this study, we constructed a novel oxidative stress-related gene prognostic signature, which shows the potential for risk stratification, prediction of prognosis and immune response in CRC patients. We identified 9 prognostic OS-related genes in CRC, and CRC patients were categorized into 2 OS-related molecular subtypes (C1, C2). The Lasso regression further selected 8 prognostic OS-related genes (STK25, CTNNB1, HSPB1, MMP3, SFPQ, RNF112, NOL3, PAGE4) and constructed a prognostic 8-gene risk model. The prognostic value of these genes, subtypes or the constructed risk signature in CRC was identified, and the association between the tumor immune cell infiltration with the C1/C2 or high-/low-risk groups was confirmed.

OS is a pathological response implicated in the development of a variety of diseases [25]. ROS levels are different in cancer cells than in normal cells, and oxidative DNA damage increases the cancer risks [26]. In this study, we identified 9 prognostic OS-associated genes in CRC patients based on univariate Cox regression analyses, including STK25, CTNNB1, HSPB1, MMP3, SFPQ, RNF112, NOL3, PAGE4, NCOA7. Previous studies have revealed that STK25 is lowly expressed in CRC tissues, and CRC patients with high STK25 expression are predicted with favorable prognosis. STK25 overexpression inhibits CRC cell autophagy by regulating the JAK2/STAT3 signaling [27]. A study also indicates that STK25 overexpression suppresses CRC cell proliferation and aerobic glycolysis in vitro, while STK25 silencing shows opposite effects on CRC cell growth [28]. CTNNB1 is a key regulator of the Wnt signaling and encodes the β-catenin 1 protein. This signaling is implicated in the regulation of tumorigenesis, stemness, TME and metabolism of various cancers, CRC included [29–31]. The stabilization of CTNNB1 by ACLY is also indicated to promote cell migration and invasiveness in colon cancer [32]. HSPB1 (HSP27) overexpression is demonstrated to reverse the anti-tumor impact of miR-214 on colon cancer cell growth and resistance to 5-FU [33]. High HSPB1 expression predicts adverse survival outcomes in CRC patients, and HSPB1 is suggested as an independent prognostic biomarker for UICC stage I/II patients [34]. MMP3 is highly expressed in the CRC tissues and is indicated to promote cancer cell migration and invasion [35, 36]. SFPQ is reported to exert oncogenic effects on CRC cell proliferation and apoptosis, and lncRNA-422 inhibits CRC cell growth by targeting SFPQ [37]. RNF112 is abundant in the brain and is indicated to play protective roles against brain injury and maintain brain functions [38, 39]. However, the biological functions of RNF112 in CRC progression are rarely reported. NOL3 is reported as an autophagy-associated gene in CRC, and patients with high levels of NOL3 have adverse clinical outcomes [40]. PAGE4 is a member of the Cancer Testis Antigen family and shows protective effects on prostate cancer cells against OS-caused cell apoptosis by attenuating DNA damage [41]. PAGE4 is also upregulated in the primary tumor samples of CRC with liver metastasis and is suggested as a potential biomarker to predict liver metastasis in CRC [42]. NCOA7 expression is higher in the low-risk colon adenocarcinoma patients and shows a negative association with the risk score in an immune-associated risk model for colon adenocarcinoma prognosis [43]. In this study, we categorized the CRC patients into C1, C2 subtypes with the consensus clustering of the OS-related genes. CRC patients in the C1 subtype had adverse survival outcomes. We also constructed a prognostic risk model based on Lasso regression, and 8 OS-related RNAs were selected in this model. Risk score was applied for categorizing CRC patients, and those with low-risk scores had more favorable overall survival outcomes. Moreover, we also found that the C2 subtype was associated with lower risk scores, which was consistent with our findings of the CRC patient prognosis. The predictive value of the 8 selected genes in prognosis was evaluated, and the ROC curves indicated that CTNNB1, STK25, RNF112, SFPQ, MMP3 and NOL3 were promising prognostic biomarkers for CRC patients. Furthermore, the correlation of the prognostic 8 OS-related RNAs with the stemness and MSI in CRC was evaluated, and a less significant association was found. We then explored the expression and functions of 8 genes in CRC and identified that CTNNB1, HSPB1, MMP3 and NOL3 were upregulated in CRC tissue samples and cells. Knockdown of CTNNB1, HSPB1, MMP3 and NOL3 hindered CRC cell proliferation and stemness and facilitated CRC cell apoptosis, which may provide novel therapeutic targets for CRC.

OS has been revealed to modulate the immune cell functions in TME, which consists of tumor constituents as well as non-tumor components such as stromal and immune cells [44]. In our work, the immune infiltration analysis revealed a strong association between CRC subtypes or risk scores with the immune response. Patients in the C1 subtype or high-risk group were related to stronger immunosuppression with lower levels of T/T-helper cells, which was consistent with previous findings [45, 46], suggesting that the lower immune activity contributed to the adverse prognosis in the C1 subtype and high-risk group of CRC patients.

With the advancement of high-through sequencing, numerous CRC-related biomarkers are identified. The underlying regulatory mechanism of the selected biomarkers requires further investigation. Li et al. have established an LMI-INGI model to predict the interactions between lncRNAs and miRNAs based on interactome network and graphlet interaction, which shows high prediction performance and applicability [47]. Another study has reported the development of the network distance analysis model for the prediction of lncRNA-miRNA interactions (NDALMA), with good prediction accuracy and suitability [48]. Wang et al. propose a GCNCRF method for the prediction of lncRNA-miRNA interactions based on graph convolutional neural (GCN) and network and conditional random field (CRF) with an AUC value of 0.947 in validation, showing higher prediction accuracy compared with the other methods [49]. Based on deep learning method, the graph convolutional network with graph attention network (GCNAT) and MDA-AENMF model based on auto-encoder and non-negative matrix factorization are developed for the predictions of associations between diseases and metabolites, and their prediction accuracy has been verified [50, 51]. Moreover, the deep learning predictive model named DMFGAM is constructed for the prediction of molecules related to cardiotoxicity with excellent performance, which provides a useful tool of the discovery and development of drugs [52]. In this study, the interaction between the OS-related genes and the underlying mechanisms of these genes were not investigated. Therefore, the effective computational prediction models are expected to be explored in future research for deepening the understanding of the potential regulatory mechanisms of the screened biomarkers.

We need to acknowledge that there are some limitations to our work. First, based on bioinformatics technology, the results were only verified in the in vitro studies, and animal experiments are needed to explore the roles of the selected prognostic OS-related genes in future studies. Second, the regulatory mechanisms of the selected oxidative-related genes in CRC were not explored. Third, the CRC patient data were only collected from the public databases and expected to be validated from other sources in the future.

In conclusion, we proposed two OS-related CRC subtypes and a prognostic risk model based on OS-related genes. The risk score or CRC subtypes was significantly associated with the immune response and CRC patient survival, and the predictive accuracy for CRC prognosis was validated. The results of this work may provide clues for the design of individualized therapeutic strategies for CRC patients.

### Supplementary Information

**Additional file 1: Figure 8.** (A) Western blot was conducted to detect the protein expression of the 8 oxidative stress-related genes in indicated groups shown in the manuscript file.

**Additional file 2.** 

## Data Availability

The datasets generated during and/or analyzed during the current study are available from the corresponding author upon reasonable request. Data are also available through GitHub repository: https://github.com/ZSK5/OS_CRC.git.

## References

[1] Sung H (2021). Global Cancer Statistics 2020: GLOBOCAN Estimates of Incidence and Mortality Worldwide for 36 Cancers in 185 Countries. CA Cancer J Clin.

[2] Dekker E (2019). Colorectal cancer. Lancet.

[3] Siegel RL, Miller KD, Jemal A (2020). Cancer statistics, 2020. CA Cancer J Clin.

[4] Siegel RL (2017). Colorectal cancer statistics, 2017. CA Cancer J Clin.

[5] Acevedo-León D (2022). Oxidative Stress and DNA Damage Markers in Colorectal Cancer. Int J Mol Sci.

[6] Basak D, Uddin MN, Hancock J (2020). The Role of Oxidative Stress and Its Counteractive Utility in Colorectal Cancer (CRC). Cancers (Basel).

[7] Tong L (2015). Reactive oxygen species in redox cancer therapy. Cancer Lett.

[8] Lu C (2020). Crosstalk of MicroRNAs and oxidative stress in the pathogenesis of cancer. Oxid Med Cell Longev.

[9] Sawai K (2022). Oxidative stress as a biomarker for predicting the prognosis of patients with colorectal cancer. Oncology.

[10] Cao Y (2021). An oxidative stress index-based score for prognostic prediction in colorectal cancer patients undergoing surgery. Oxid Med Cell Longev.

[11] Liu Q, Yu M, Zhang T (2022). Construction of oxidative stress-related genes risk model predicts the prognosis of uterine corpus endometrial cancer patients. Cancers (Basel).

[12] Dong C, Zhang N, Zhang L (2021). The multi-omic prognostic model of oxidative stress-related genes in acute myeloid leukemia. Front Genet.

[13] Liu Q (2022). Identifying the role of oxidative stress-related genes as prognostic biomarkers and predicting the response of immunotherapy and chemotherapy in ovarian cancer. Oxid Med Cell Longev.

[14] Wang X (2022). A novel oxidative stress- and ferroptosis-related gene prognostic signature for distinguishing cold and hot tumors in colorectal cancer. Front Immunol.

[15] Chen Z (2022). Prognostic assessment of oxidative stress-related genes in colorectal cancer and new insights into tumor immunity. Oxid Med Cell Longev.

[16] Kennel KB, Greten FR (2021). Immune cell - produced ROS and their impact on tumor growth and metastasis. Redox Biol.

[17] Weinberg F, Ramnath N, Nagrath D (2019). Reactive oxygen species in the tumor microenvironment: an overview. Cancers (Basel).

[18] Kotsafti A (2020). Reactive oxygen species and antitumor immunity-from surveillance to evasion. Cancers (Basel).

[19] Yu G (2012). clusterProfiler: an R package for comparing biological themes among gene clusters. OMICS.

[20] Xu T (2017). CancerSubtypes: an R/Bioconductor package for molecular cancer subtype identification, validation and visualization. Bioinformatics.

[21] Hänzelmann S, Castelo R, Guinney J (2013). GSVA: gene set variation analysis for microarray and RNA-seq data. BMC Bioinformatics.

[22] Bindea G (2013). Spatiotemporal dynamics of intratumoral immune cells reveal the immune landscape in human cancer. Immunity.

[23] Lei X (2020). Immune cells within the tumor microenvironment: Biological functions and roles in cancer immunotherapy. Cancer Lett.

[24] Chandimali N, Jeong DK, Kwon T (2018). Peroxiredoxin II regulates cancer stem cells and stemness-associated properties of cancers. Cancers (Basel).

[25] Forman HJ, Zhang H (2021). Targeting oxidative stress in disease: promise and limitations of antioxidant therapy. Nat Rev Drug Discov.

[26] Hayes JD, Dinkova-Kostova AT, Tew KD (2020). Oxidative stress in cancer. Cancer Cell.

[27] Chen J (2022). Downregulation of STK25 promotes autophagy via the Janus kinase 2/signal transducer and activator of transcription 3 pathway in colorectal cancer. Mol Carcinog.

[28] Wu F (2018). STK25-induced inhibition of aerobic glycolysis via GOLPH3-mTOR pathway suppresses cell proliferation in colorectal cancer. J Exp Clin Cancer Res.

[29] Zhang L (2020). CircAGFG1 drives metastasis and stemness in colorectal cancer by modulating YY1/CTNNB1. Cell Death Dis.

[30] Tang Q (2020). TM4SF1 promotes EMT and cancer stemness via the Wnt/β-catenin/SOX2 pathway in colorectal cancer. J Exp Clin Cancer Res.

[31] Zhu Y (2020). LINC00365 promotes colorectal cancer cell progression through the Wnt/β-catenin signaling pathway. J Cell Biochem.

[32] Wen J (2019). ACLY facilitates colon cancer cell metastasis by CTNNB1. J Exp Clin Cancer Res.

[33] Yang Y (2019). MiR-214 sensitizes human colon cancer cells to 5-FU by targeting Hsp27. Cell Mol Biol Lett.

[34] Bauer K (2012). High HSP27 and HSP70 expression levels are independent adverse prognostic factors in primary resected colon cancer. Cell Oncol (Dordr).

[35] Yu J (2021). Comprehensive analysis of the expression and prognosis for MMPS in human colorectal cancer. Front Oncol.

[36] Wen Y (2022). Histone deacetylase (HDAC) 11 inhibits matrix metalloproteinase (MMP) 3 expression to suppress colorectal cancer metastasis. J Cancer.

[37] Meng Y (2022). LncRNA-422 suppresses the proliferation and growth of colorectal cancer cells by targeting SFPQ. Clin Transl Med.

[38] Zhang F, Zhang C (2018). Rnf112 deletion protects brain against intracerebral hemorrhage (ICH) in mice by inhibiting TLR-4/NF-κB pathway. Biochem Biophys Res Commun.

[39] Tsou JH (2017). Important roles of ring finger protein 112 in embryonic vascular development and brain functions. Mol Neurobiol.

[40] He Q (2021). Prognostic significance of autophagy-relevant gene markers in colorectal cancer. Front Oncol.

[41] Lv C (2019). PAGE4 promotes prostate cancer cells survive under oxidative stress through modulating MAPK/JNK/ERK pathway. J Exp Clin Cancer Res.

[42] Chen Z (2010). Cancer/testis antigens and clinical risk factors for liver metastasis of colorectal cancer: a predictive panel. Dis Colon Rectum.

[43] Lu J (2022). Establishment and evaluation of module-based immune-associated gene signature to predict overall survival in patients of colon adenocarcinoma. J Biomed Sci.

[44] Augustin RC, Delgoffe GM, Najjar YG (2020). Characteristics of the tumor microenvironment that influence immune cell functions: hypoxia, oxidative stress, metabolic alterations. Cancers (Basel).

[45] Zhu J (2018). T helper cell differentiation, heterogeneity, and plasticity. Cold Spring Harb Perspect Biol.

[46] Dong C (2021). Cytokine regulation and function in T cells. Annu Rev Immunol.

[47] Zhang L (2021). Predicting lncRNA-miRNA interactions based on interactome network and graphlet interaction. Genomics.

[48] Zhang L (2021). Using network distance analysis to predict lncRNA-miRNA interactions. Interdiscip Sci.

[49] Wang W (2022). Predicting the potential human lncRNA-miRNA interactions based on graph convolution network with conditional random field. Brief Bioinform.

[50] Sun F, Sun J, Zhao Q (2022). A deep learning method for predicting metabolite-disease associations via graph neural network. Brief Bioinform.

[51] Gao H (2023). Predicting metabolite-disease associations based on auto-encoder and non-negative matrix factorization. Brief Bioinform.

[52] Wang T, Sun J, Zhao Q (2023). Investigating cardiotoxicity related with hERG channel blockers using molecular fingerprints and graph attention mechanism. Comput Biol Med.

