# Supplementary material for: Prognostic value of oxidative stress-related genes in colorectal cancer and its correlation with tumor immunity
Source: BMC Genomics. 2024 Jan 2;25:8. doi: 10.1186/s12864-023-09879-0 (PMC10759670; doi:10.1186/s12864-023-09879-0)
Supplement: Supplementary file 1 — Additional file 1: Figure 8. (A) Western blot was conducted to detect the protein expression of the 8 oxidative stress-related genes in indicated groups shown in the manuscript file. [file 12864_2023_9879_MOESM1_ESM.docx]

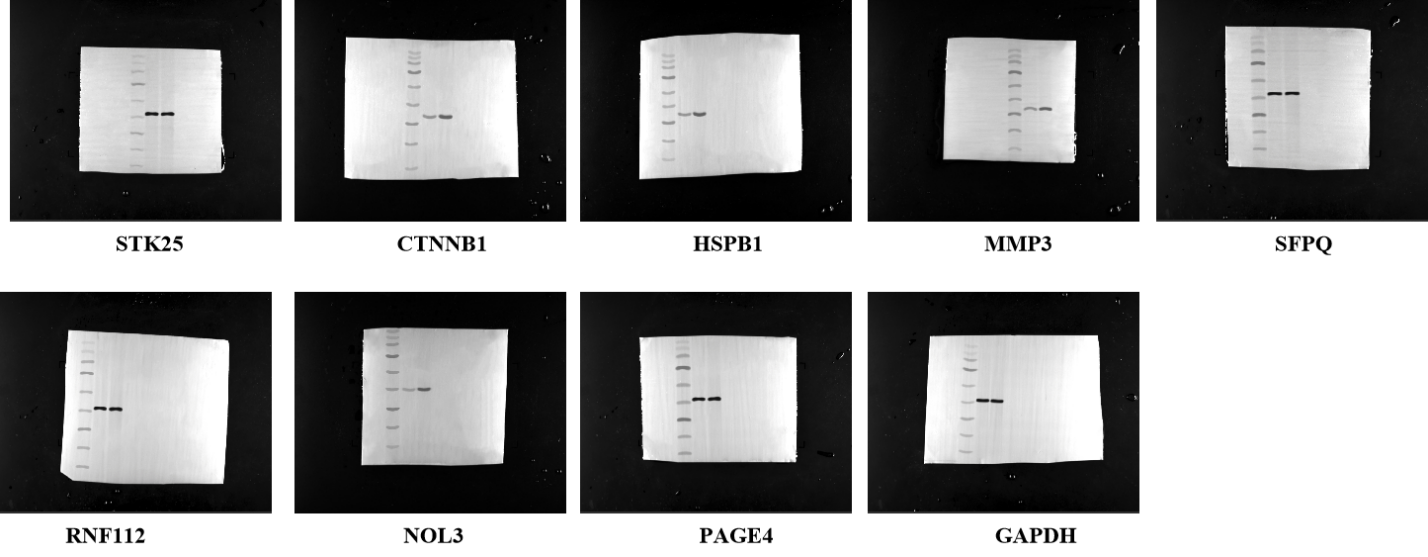


Figure 8. (A) Western blot was conducted to detect the protein expression of the 8 oxidative stress-related genes in indicated groups shown in the manuscript file.
