# Supplementary figures and images for: Prognostic value of oxidative stress-related genes in colorectal cancer and its correlation with tumor immunity
Source: BMC Genomics. 2024 Jan 2;25:8. doi: 10.1186/s12864-023-09879-0 (PMC10759670; doi:10.1186/s12864-023-09879-0)

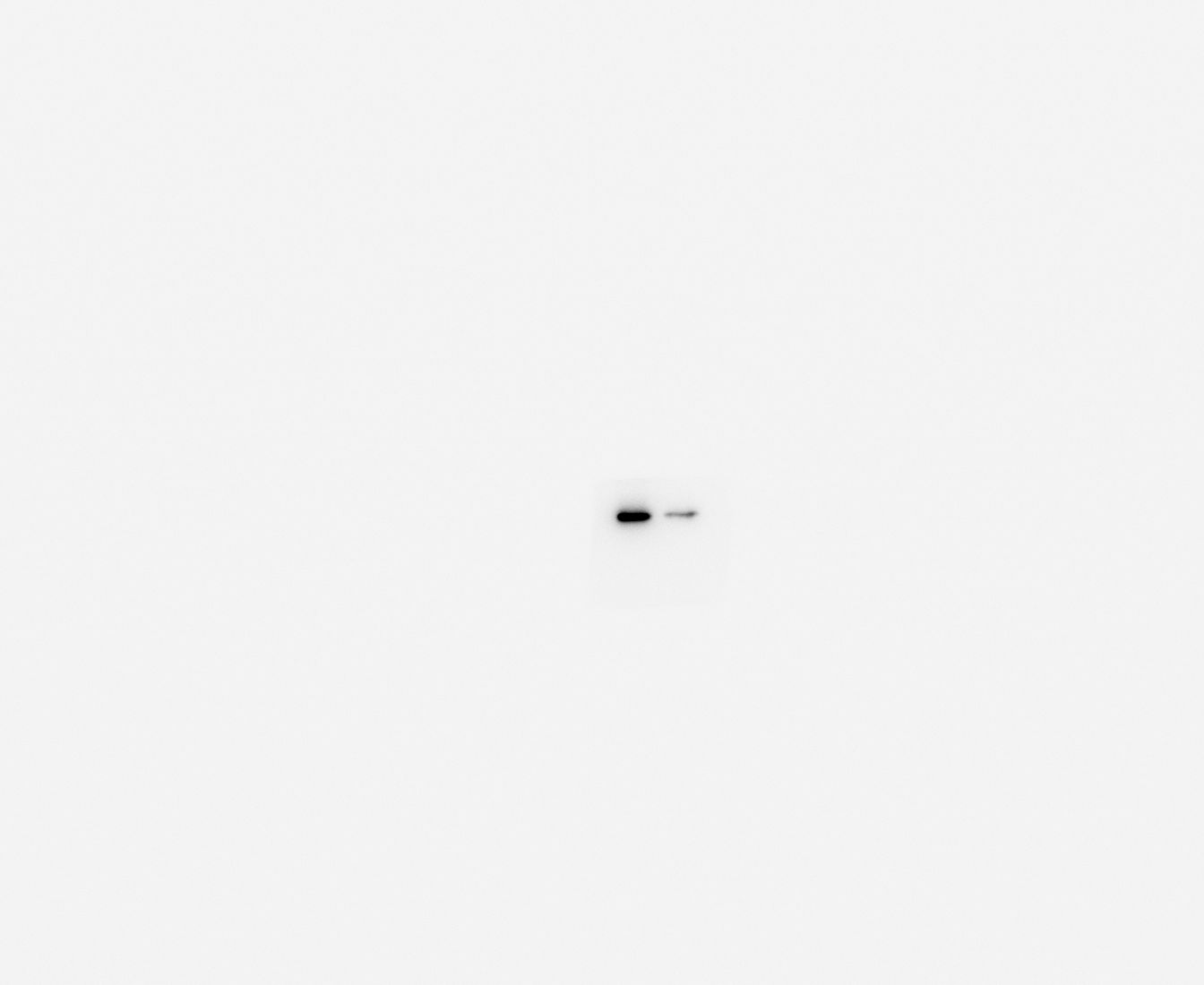

Supplement: Supplementary file 2 — Additional file 2. [file 12864_2023_9879_MOESM2_ESM.zip › Supplementary material/Supplementary material/WB/CTNNB1.tif]

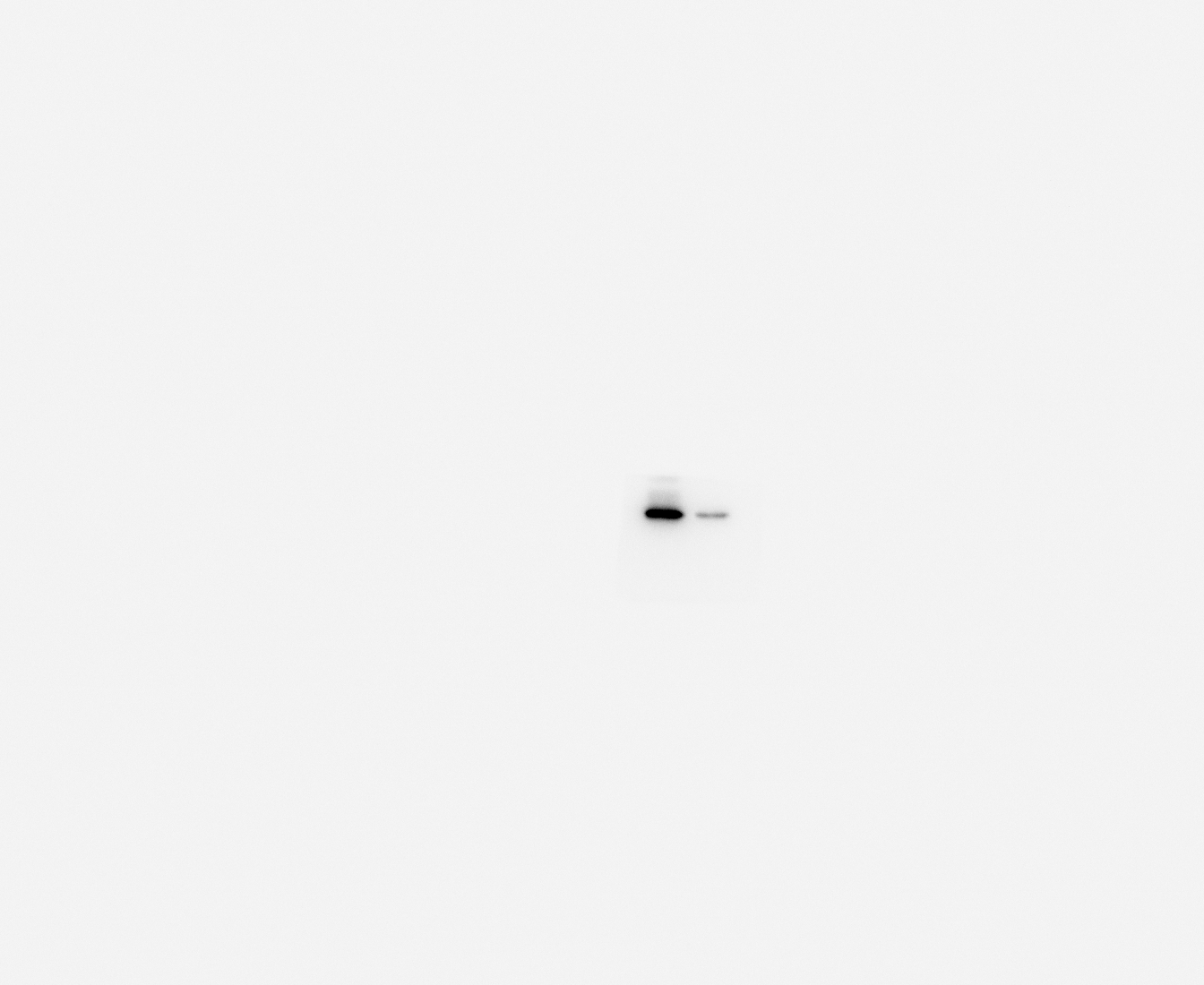

Supplement: Supplementary file 2 — Additional file 2. [file 12864_2023_9879_MOESM2_ESM.zip › Supplementary material/Supplementary material/WB/HSPB1.tif]

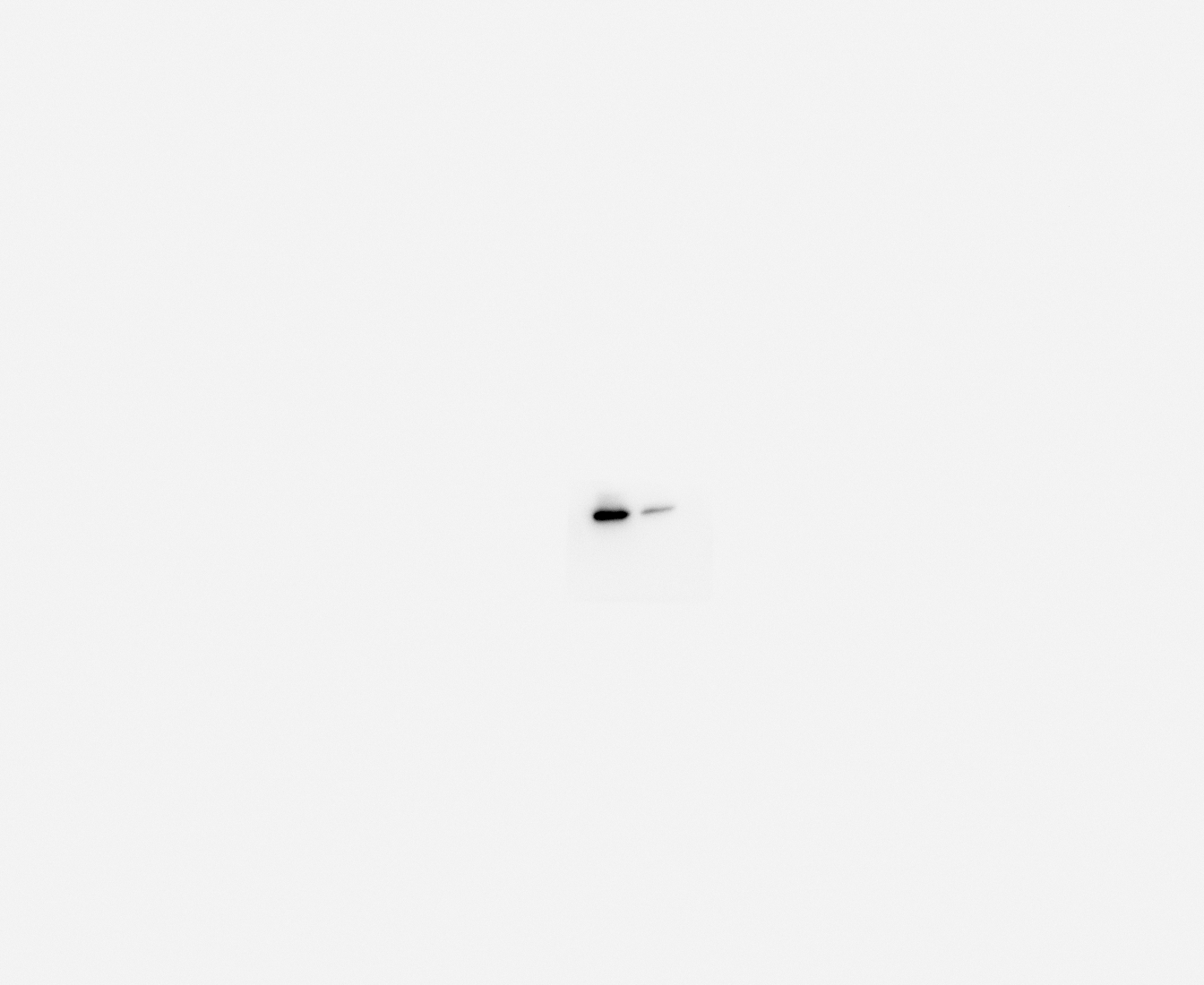

Supplement: Supplementary file 2 — Additional file 2. [file 12864_2023_9879_MOESM2_ESM.zip › Supplementary material/Supplementary material/WB/MMP3.tif]

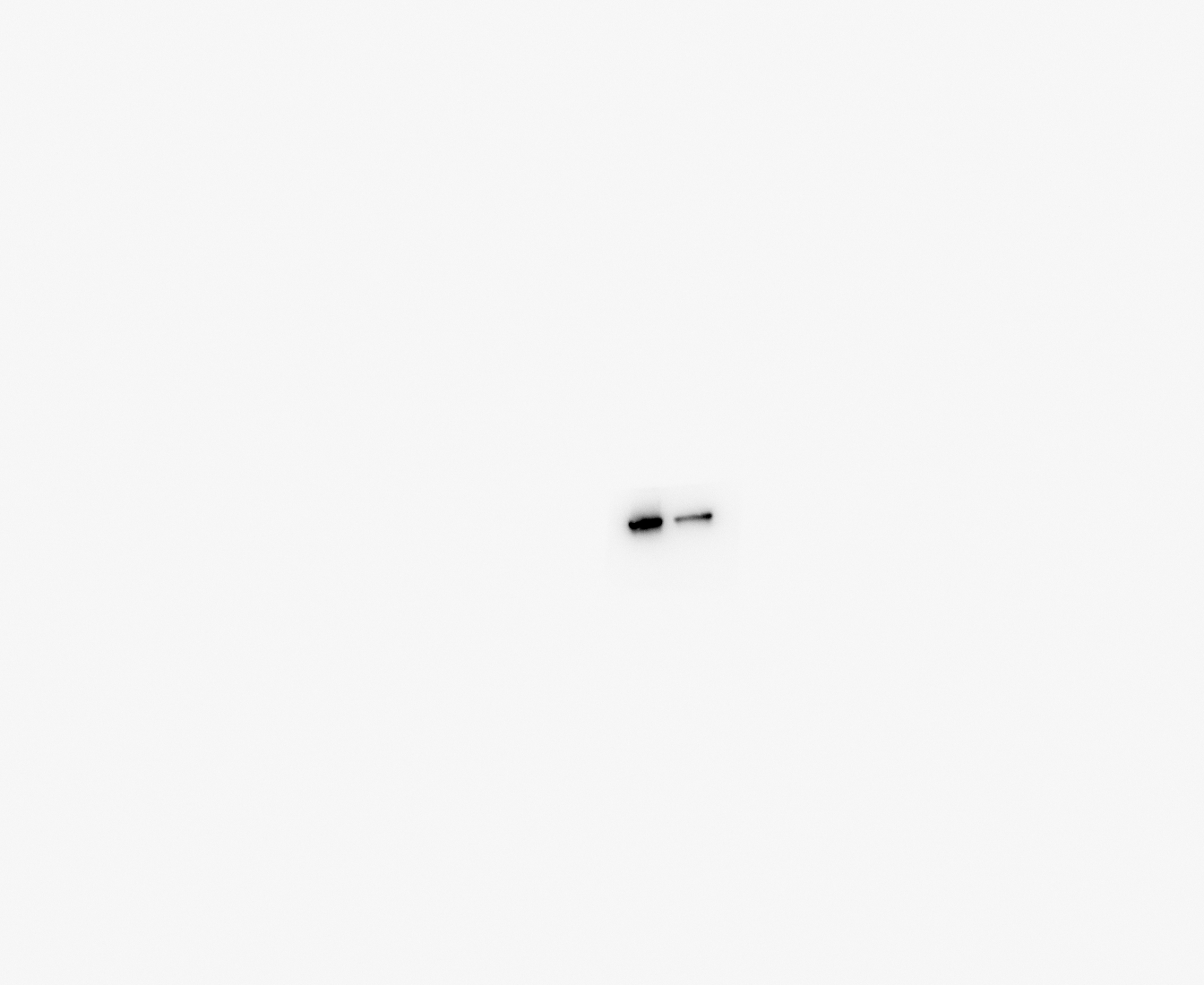

Supplement: Supplementary file 2 — Additional file 2. [file 12864_2023_9879_MOESM2_ESM.zip › Supplementary material/Supplementary material/WB/NOL3.tif]

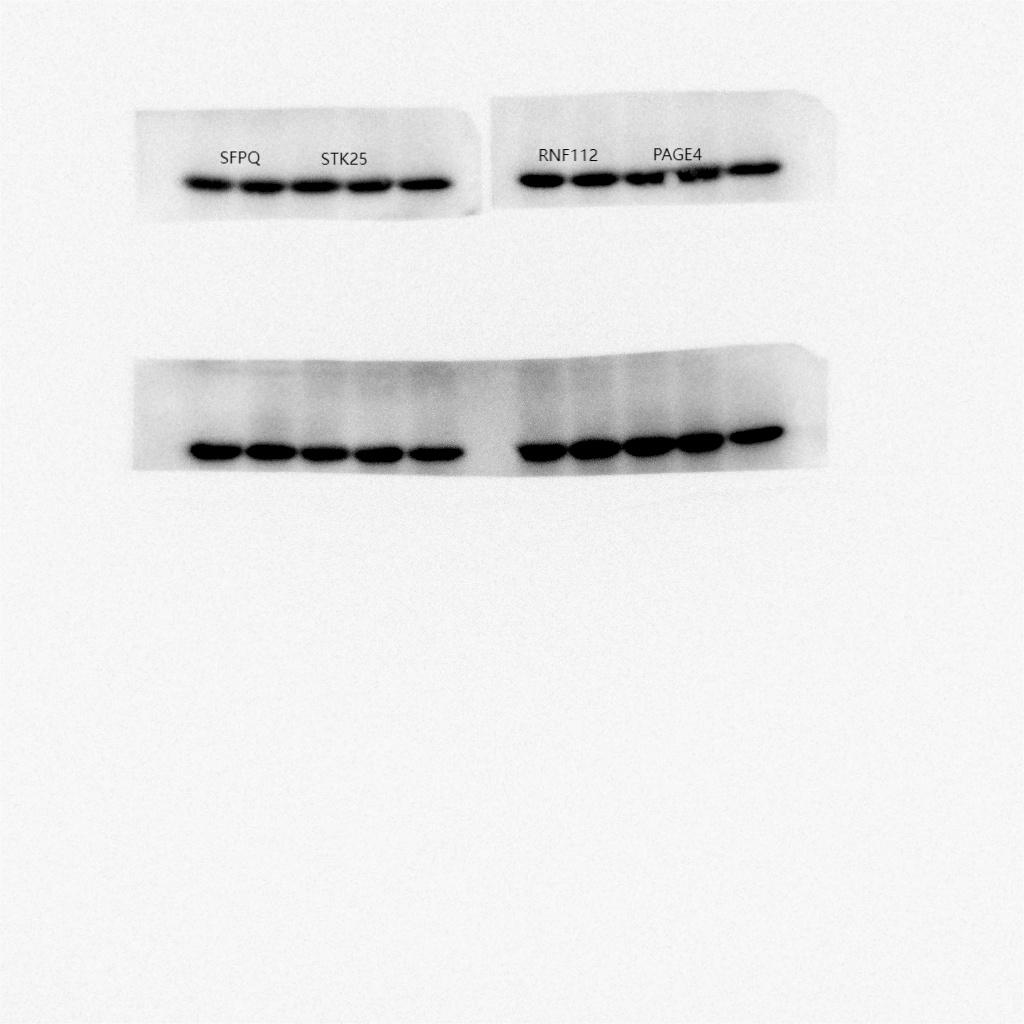

Supplement: Supplementary file 2 — Additional file 2. [file 12864_2023_9879_MOESM2_ESM.zip › Supplementary material/Supplementary material/WB/SFPQ,STK25,RNF112,PAGE4.jpg]
